# The Impact of Anxiety and Depressive Symptomatology on Greek Practicing Lawyers’ Self-Esteem

**DOI:** 10.3390/healthcare12232428

**Published:** 2024-12-03

**Authors:** Andreas-Nikolaos Koukoulis, Melissa Theocharidou, Emmanouil-Ioannis Kallergis, Olympia Kosta, Stavroula Papadodima, Maria Tsellou

**Affiliations:** 1 Court of First Instance of Athens, Former Evelpidon School, 11362 Athens, Greece; 2 Independent Researcher, 54621 Thessaloniki, Greece; 3 Independent Researcher, 12131 Athens, Greece; 4 Court of Appeal of Athens, 14 Kyrillou Loukareos Street, Kountouriotika, 11522 Athens, Greece; 5 Department of Forensic Medicine and Toxicology, School of Medicine, National and Kapodistrian University of Athens, M. Asias 75, 11527 Athens, Greece; stpapd@med.uoa.gr (S.P.); mtsellou@med.uoa.gr (M.T.)

**Keywords:** anxiety, depression, self-esteem, lawyers, mental health, Greek legal professionals, Beck Anxiety Inventory (BAI), Beck Depression Inventory (BDI), Rosenberg Self-Esteem Scale (RSES), psychological well-being

## Abstract

Background/Objectives: This study examines the impact of anxiety and depressive symptomatology on self-esteem among Greek practicing lawyers. The high-stress environment of the legal profession is known to heighten the risk of mental health issues, yet limited research exists on Greek lawyers. The study aims to explore associations between anxiety, depressive symptoms, and self-esteem, hypothesizing that increased anxiety and depression correlate with reduced self-esteem. Method: The study utilized a correlational design with a sample of 80 Greek lawyers, recruited through professional associations. Participants completed the Beck Anxiety Inventory (BAI), Beck Depression Inventory (BDI), and Rosenberg Self-Esteem Scale (RSES). Descriptive statistics, correlation analysis, and multiple regression were used to assess the relationships between anxiety, depression, and self-esteem. Results: The findings indicated significant negative correlations between self-esteem and both depressive (*r* = −0.631, *p* < 0.001) and anxiety symptoms (*r* = −0.405, *p* < 0.001). Multiple regression analysis demonstrated that both depression (*β* = −0.558, *p* < 0.001) and anxiety (*β* = −0.225, *p* = 0.014) were significant predictors of lower self-esteem, explaining approximately 44% of the variance in self-esteem scores. Conclusions: The study concludes that higher levels of anxiety and depressive symptoms significantly lower self-esteem among Greek lawyers, underscoring the need for mental health support within this profession. Addressing mental health issues in legal professionals may promote better self-esteem and overall well-being, with potential benefits for both lawyers and their clients.

## 1. Introduction

### 1.1. Anxiety

Anxiety disorders are the most prevalent mental health conditions, characterized by a complex mix of negative emotions and cognitive processes. Unlike fear, anxiety is future-oriented and encompasses feelings of worry, self-preoccupation, and a sense of helplessness regarding potential threats. It often leads to chronic overarousal and risk assessment, motivating individuals to avoid potentially risky situations without the immediate flight response seen in fear [1,2]. Genetic factors, such as neuroticism, and classical conditioning contribute to anxiety disorders, suggesting that both hereditary predispositions and learned behaviors play significant roles [3,4,5].

### 1.2. Depression

Depression can arise from significant life events such as bereavement or relationship breakdowns and varies in severity from mild to debilitating. Symptoms include prolonged feelings of sadness (dysphoria), anhedonia (loss of interest), changes in appetite and sleep patterns, and cognitive difficulties [6]. The distinction between normal grief and clinical depression remains contentious, especially in the context of recent DSM-5 guidelines [7]. Hallucinations and delusions may occur in severe cases [8].

### 1.3. Anxiety-Depression Link

Anxiety and depression frequently coexist and share common underlying traits, such as a tendency to ruminate on past, present, and future worries. Trait-anxiety has been identified as a risk factor for developing depression, with many individuals experiencing both conditions at different stages in life [8,9,10,11]. Personality characteristics can influence resilience and vulnerability to these emotional disorders [12].

### 1.4. Self-Esteem

High self-esteem serves as a vital psychosocial resource, effectively reducing feelings of depression and anxiety and protecting psychological well-being from stress [13,14]. Individuals with high self-esteem often demonstrate improved mental and physical health, which can result from successfully managing anxiety, depression, and stress [15]. Conversely, those with low self-esteem are at a higher risk for developing depression [16].

Significant research links high levels of anxiety to an increased likelihood of depression [17,18,19]. Studies indicate that a positive emotional impact with minimal adverse effects is beneficial for self-esteem, and various psychological adjustment markers, such as high positive affect and low negative affect, correlate with elevated self-esteem [20,21,22,23]. However, the intricate interactions between these variables remain complex and not fully understood [24].

Low self-esteem can lead to feelings of hopelessness, contributing to a specific type of depression known as hopelessness depression [25]. Interestingly, effectively managed stressful events can foster self-confidence and resilience, suggesting that moderate stressors may ironically enhance coping abilities [2]. Attributes such as optimism, mental strength, and social support are associated with improved resilience during stressful situations [26,27].

However, inflated self-esteem can act as a defense mechanism, preventing individuals from addressing deeper feelings of low self-worth or self-pity [28]. Negative thinking patterns often correlate with inadequate self-worth [29].

The notion of self-esteem has faced scrutiny, particularly concerning educational initiatives aimed at raising children’s self-worth without linking it to actual accomplishments. Critics argue that promoting self-esteem independently of real achievement may not be effective [30]. Consequently, high self-esteem appears to be both a result and a cause of success; efforts to boost self-esteem without tangible accomplishments seem insufficient [24]. Additionally, while low self-esteem is closely associated with depression and other behavioral issues, the underlying reasons for this connection require further exploration [14].

Generally, it seems that the relationship between self-esteem and psychological well-being is intricate, as both elevated and diminished levels of self-esteem can contribute to the emergence of behavioral issues.

In the present research, we focus on the impact of anxiety and depressive symptomatology on practicing lawyers’ self-esteem. There is research focusing on other target groups, like patients with Polycystic ovary syndrome (PCOS) [31], individuals with cardiovascular diseases [32], young adults [33], couples undergoing infertility treatment [34], university students [35], adult acquired brain injury [36], older people in nursing homes [37], and migrant populations [38]. There is no research referring to lawyers and it is important to find out how these professionals react to anxiety and depressive symptoms.

This research is important if we think the following: Assume we assert that low self-esteem leads to and sustains depression. This assertion predicts that people who have depression have less confidence than non-depressed patients. If we evaluate the two types of patients and discover that the depressed team ratings are significantly lower than those of the non-depressed team, we could have provided some backing for the hypothesis, but we cannot demonstrate it is correct: another, as yet unresolved aspect may cause depression as well as low self-esteem. These findings, nevertheless, enable us to dismiss the null hypothesis, which states that there are no changes in self-esteem among depressed and non-depressed teams. This excludes at least one additional conceivable hypothesis: that poor self-esteem is indicative of everyone, no matter if they are experiencing depression [39]. People suffering from social anxiety frequently have a lack of self-worth and are predisposed to mood disorders such as depression [40].

The legal profession is often characterized by high levels of stress, stemming from the adversarial nature of litigation, strict deadlines, and the constant need to meet client expectations. Lawyers must navigate a complex professional environment that includes long working hours, emotionally demanding cases, and significant responsibility for client outcomes [41]. Research has consistently demonstrated that these stressors contribute to elevated levels of anxiety and depression among legal professionals, negatively impacting their self-esteem and overall well-being [42,43,44].

In Greece, the intersection of economic challenges, cultural expectations, and the nature of legal work creates a unique landscape for understanding the mental health of lawyers. Furthermore, lawyers in Greece face limited career mobility and systemic inefficiencies within the judiciary. These factors exacerbate stress levels and underscore the need to examine mental health in this professional group. The financial crisis has had lasting effects on the legal profession, leading to increased workloads, job insecurity, and heightened stress levels. Additionally, societal stigma surrounding mental health may hinder open discussions about these issues, preventing lawyers from seeking necessary support. Despite extensive research on mental health in other high-stress professions, studies specifically focusing on Greek lawyers remain sparse. Given the unique stressors they encounter, understanding how anxiety and depression impact their self-esteem is critical.

Recent studies emphasize the interplay between mental health and self-esteem, particularly in high-stress occupations. For example, findings suggest that remote work, economic pressures, and evolving professional practices have intensified mental health challenges in professions such as healthcare and corporate leadership [45,46,47,48]. This study aims to fill a critical gap in the literature by exploring the associations between anxiety, depressive symptomatology, and self-esteem among Greek lawyers. It also highlights the need for tailored interventions to support their mental well-being.

Before disentangling the impact of anxiety and depressive symptomatology on practicing lawyers’ self-esteem in the current literature, we briefly present the proposed methodology that will be used in this study.

### 1.5. Aims and Hypothesis

The mental health of legal professionals has garnered significant attention due to the high-stress nature of the profession. Lawyers often face intense pressure, which can lead to various mental health issues, including anxiety and depression. These conditions can significantly impact their professional and personal lives. In the present research, we focus on the impact of anxiety and depressive symptomatology on practicing lawyers’ self-esteem. There are researches focusing on other target groups, like individuals with cardiovascular diseases where the prevalence of depression and anxiety symptoms was reported at 32.5% and 17.5%, respectively, with notable differences related to gender [32]. Notably, symptoms of depression have been linked to the prevalence of comorbidities. Individuals who recovered more quickly from cardiovascular issues were those who did not exhibit depression symptoms; interestingly, women who expressed worry tended to recover at a faster rate. Patients suffering from anxiety and depression had the highest self-esteem ratings. Men demonstrated more stamina and less self-confidence than women and young adults [33]. The findings imply that initial therapy for depression in young people should focus on increasing their sense of social support, adaptability, and beneficial personality characteristics. More research on the impact of high self-esteem on the onset of depression is needed in couples undergoing infertility treatment [34]. Women reported greater emotional suffering than men in a variety of symptom areas, including generalized mental disorders, anxiety, depression, and self-worth. In adults with acquired brain injuries (ABI), low self-esteem is associated with emotional disorders, maladaptive coping mechanisms, and reduced community involvement [36]. For older adults in care facilities, research has identified significant correlations between self-esteem, anxiety, and depression. In the realm of sports, sport-related anxiety has been found to correlate closely with certain types of perfectionism, particularly among individuals employing specific self-esteem strategies [49]. Furthermore, anxiety is a significant factor influencing the perceived dental effects on self-esteem in mature orthodontic patients [50]. In the context of domestic violence, it has been observed that symptoms of depression among battered women are correlated with a decline in self-esteem [51]. Another notable finding is the significant relationship between severe burns and increased levels of anxiety and depression, alongside diminished self-worth [52]. There is no research referring to Greek lawyers and it is important to find out how these professionals react to anxiety and depressive symptoms. The American Bar Association has released troubling data: 28% of lawyers in the United States are depressed, 19% are anxious, and 21% are alcoholics [42]. Previous studies have highlighted the prevalence of mental health issues among lawyers. Beck et al. 1995 [53] conducted seminal studies on lawyer distress, revealing high levels of anxiety and depression in this population. Additionally, studies on different populations, such as PCOS patients [31], have shown similar correlations between mental health and self-esteem. However, there is a gap in the literature regarding Greek lawyers, necessitating this study.

This study aims to explore the associations between anxiety, depressive symptomatology, and self-esteem among Greek practicing lawyers. We hypothesize that:

1. There is a significant association between anxiety levels and self-esteem.
2. There is a significant association between depressive symptomatology and self-esteem.
3. These associations will provide insights into the mental health challenges faced by Greek lawyers.

## 2. Materials and Methods

### 2.1. Participants

Participants were recruited via email. Email addresses were obtained through professional legal associations in Greece. A total of about 800 lawyers were invited to participate, with follow-up emails sent to increase the response rate. The recruitment took place from November 2020 to August 2022. Participation was anonymous to ensure candid responses. The participants included those of all genders (males, females, non-binary, other, and prefer not to say), and were aged 18 or above.

### 2.2. Design

This study focused on the relationship between anxiety and depressive symptomatology and self-esteem in practicing lawyers. Therefore, the design of this study was a correlational design. The predictor variables were the self-esteem of practicing lawyers, and the criterion variables were anxiety and depressive symptomatology.

### 2.3. Materials

These resources were made available via the college as well as research publications (including any questionnaires used). The survey included the following standardized instruments:

- “The Rosenberg Self-Esteem Scale (RSES)”: A widely used measure of self-esteem with proven reliability and validity.
- “Beck Anxiety Inventory (BAI)”: A 21-item self-report inventory measuring the severity of anxiety, known for its accuracy and sensitivity.
- “Beck Depression Inventory (BDI)”: A 21-item self-report inventory assessing the severity of depression, with established sensitivity and specificity.

Additional questions assessed demographic information and musculoskeletal pain, though these were not the primary focus of this analysis.

### 2.4. Procedure

Participants read through the participant information online form and provided consent for data collection before starting the project (Appendix A). At the start of the survey, participants read the participant data page, which included all necessary details about the research they were about to engage in and provided researcher contact information in case they required further assistance or had any concerns. Participants were notified of the research anonymity and their opportunity to opt out by not completing their responses. They were also informed that they would not be able to withdraw after submitting their answers, as the study was online and anonymous, making it impossible to trace data back to specific participants. The participants then completed the consent form, where all criteria needed to be met for them to participate in the study. This study required each participant to respond to the Beck Anxiety Inventory (BAI), the Beck Depression Inventory (BDI) (except for question number 9), and the Rosenberg Self-Esteem Scale. After completing the survey, participants were debriefed about the study and thanked for their participation. For ethical considerations, it was stated to the participants that the research concerned depressive and anxiety symptomatology and not disorders. At the end of the questionnaire, it was indicated that if participants had any questions, they should use the contact details provided at both the start and the end of the questionnaire. Contact details for a local mental health center were also given in case of distress. The study was expected to take around 20 min.

### 2.5. Statistical Analysis

Data were analyzed using descriptive statistics and correlation coefficients to explore associations between variables. Missing data were handled using multiple imputation. Multiple regression analyses were conducted, including anxiety and depression as independent variables, to examine their association with self-esteem. Variance Inflation Factor (VIF) assessed collinearity between the independent variables. A multiple regression analysis was conducted to investigate whether anxiety and depressive symptomatology had an effect on the self-esteem of practicing lawyers. The predictor variables were anxiety and depressive symptomatology, and the criterion variable was the self-esteem of practicing lawyers.

## 3. Results

### 3.1. Descriptive Findings

A total of 80 lawyers participated in the study (10% response rate). The sample included a diverse range of ages, genders, and years of practice. The participants’ scores on the Rosenberg Self-Esteem Scale (RSES) exhibited a mean of 27.75 (SD = 5.38) (Table 1). On the Beck Depression Inventory (BDI), the mean score was 18.01 (SD = 13.85), and for the Beck Anxiety Inventory (BAI), the mean score was 12.81 (SD = 11.07) (Table 1).

#### 3.1.1. Correlation Analysis

Pearson’s correlation analysis revealed significant associations among the psychological measures. Specifically, a significant negative correlation was observed between RSES and BDI scores, r(78) = −0.631, *p* < 0.001, indicating that higher levels of depressive symptomatology were associated with lower self-esteem scores. Additionally, a significant negative correlation was found between RSES and BAI scores, r(78) = −0.405, *p* < 0.001, suggesting that increased anxiety symptomatology was similarly associated with lower levels of self-esteem. Finally, a significant positive correlation was identified between BDI and BAI scores, r(78) = 0.322, *p* = 0.002, indicating that participants with higher depression scores also tended to report higher anxiety scores. Correlation analysis revealed significant associations between anxiety and self-esteem (r = −0.45, *p* < 0.01), and between depression and self-esteem (r = −0.60, *p* < 0.01).

#### 3.1.2. Multiple Regression Analysis

To understand how depressive and anxious symptomatology together predict self-esteem, a multiple regression analysis was performed with RSES as the dependent variable and BDI and BAI scores as predictors. (Table 2) The model was significant, F(2, 77) = 30.659, *p* < 0.001, accounting for approximately 44.3% of the variance in RSES scores, R^2^ = 0.443. The adjusted R^2^ value was slightly lower at 0.429, accounting for the number of predictors. Both BDI (*β* = −0.558, *p* < 0.001) and BAI (*β* = −0.225, *p* = 0.014) emerged as significant predictors in the model. Multiple regression analysis showed that both anxiety (*β* = −0.30, *p* < 0.05) and depression (*β* = −0.50, *p* < 0.01) were significantly associated with lower self-esteem, controlling for other variables. VIF values were within acceptable limits, indicating no significant collinearity.

### 3.2. Summary of Findings

The results suggest that, among practicing lawyers, both depressive and anxious symptomatology are significant negative predictors of self-esteem. The regression model was robust, explaining about 44% of the variance in self-esteem scores. The strongest predictor was depressive symptomatology, followed by anxiety symptomatology. These findings may have important implications for understanding the mental health challenges faced by legal professionals, especially given the significant associations among depression, anxiety, and self-esteem.

## 4. Discussion

The purpose of the present study was to examine the impact of anxiety and depressive symptomatology on practicing lawyer’ s self-esteem. This study found significant associations between anxiety, depressive symptomatology, and self-esteem among Greek lawyers. These findings align with previous research indicating high levels of mental health issues in the legal profession. The results support the alternative hypotheses, demonstrating a negative relationship between self-esteem and depressive symptomatology, as well as between self-esteem and anxiety symptomatology, among practicing lawyers. Furthermore, depressive and anxiety symptomatology were found to predict self-esteem, with higher levels of symptomatology being associated with lower levels of self-esteem. Based on the results of the multiple regression analysis, it can be concluded that both anxiety and depressive symptomatology have a significant negative impact on the self-esteem of practicing lawyers. Specifically, higher levels of anxiety and depression are associated with lower levels of self-esteem. This is supported by the negative standardized coefficients for both BAI and BDI in the model. (Table 3) In conclusion, these findings suggest that addressing anxiety and depressive symptomatology may be important in promoting self-esteem and well-being in practicing lawyers. Compared to U.S. and Polish lawyers, Greek lawyers in this study showed similar levels of anxiety and depression [53,54]. This highlights the universal nature of these issues across different legal systems.

A considerable proportion of practicing lawyers are suffering from serious mental health symptoms that exceed what would be anticipated in the general population at large. Unexpectedly, the basic structure of discomfort could point to the attributes required to succeed as an effective attorney (obsessive–compulsiveness, emotional awareness, and anxiety) as well as the consequences associated with achievement (depression, social estrangement, and solitude) [42]. Being regarded as a lawyer, particularly as a litigious lawyer, entails a lot of in-person intimate interaction and conflict. Furthermore, in order to avoid jeopardizing their clients’ privacy and security, lawyers must maintain complete focus on their duties throughout all certain points. Furthermore, with increased expertise prerequisites for non-litigious issues that are international in nature, such as corporate mergers, transfer of technology, and intellectual property, as well as increasing rivalry between lawyers in the job marketplace, which has witnessed the number of licensed lawyers rise from less than 30 per year prior to 1989 to more than 200 per year today, the professional setting for lawyers has shifted substantially. Lawyers face higher work anxiety as a result of enhanced efforts and decreasing incentives [55]. Previous study has revealed that law students have high levels of anxiety and depression beginning in their initial year of law school [56]. According to studies of practicing lawyers, most report high levels of job fulfillment and overall well-being, particularly among more senior lawyers and those employed by smaller firms [57]. A worrisome number of freshly practicing lawyers describe a wide range of substantial emotional distress symptoms, far exceeding what would be anticipated in the general population [53]. The degree of anxiety has been found to be nearly a full percentage point over the average for the population in the research of lawyers in Washington State; a little over one fifth of the group studied had rates that classified them in the top two percent of those in the general population. Lawyers had higher levels of depression and alcohol usage [53]. According to one research study of more than one hundred occupations, lawyers had among the greatest prevalence of depression [58]. Lawyers attempt suicide at a sixfold greater frequency than the overall community [59]. Depression and anxiety-related conditions appeared to be more common in Polish lawyers than in the population as a whole, and the frequency of physical manifestations rose as the degree of severity of these illnesses worsened [60]. One of the causes of serious depressive disorders among lawyers is an upsurge in the sheer quantity of lawyers, which has probably resulted in increasing competitiveness and deterioration of personal connections among other lawyers. Furthermore, today’s legal system is incredibly complex. Updated legal norms have rendered it hard to provide guidance to clients, and courts produce such numerous rulings that it is impossible for one to comprehend exactly what the law actually is. No matter what the cause is, lawyers experience greater levels of burnout, dissatisfaction and discontentment, which may give rise to disregard by attorneys of files, depression, anxiety, drug or alcohol use, or self-harm [61]. The mental health challenges faced by lawyers can negatively impact their professional performance and client relationships. For instance, lawyers with low self-esteem may struggle with effective client communication and decision making. Addressing these issues is crucial for the well-being of both lawyers and their clients. Impaired mental health in lawyers can lead to suboptimal legal outcomes, affecting clients and the broader judicial system. For example, low self-esteem in divorce lawyers can exacerbate the stress experienced by divorcing spouses and children. The first hypothesis was that there is a negative relationship between self-esteem and depression symptomatology among practicing lawyers. A survey of 7551 Australian practitioners discovered that lawyers had a significantly greater frequency of symptoms of depressive disorders when weighed against nine other occupations, with 15.2% having medium to serious symptoms of depression, opposed to an average for all occupations of 10.5% [62]. According to our research, the results of a Beck Anxiety Inventory scoring of Greek practicing lawyers, 76.25% had low anxiety symptomatology, 17.50% had moderate anxiety, and 6.25% had potentially concerning levels. According to Beck Depression Inventory (BDI), 40.00% were normal, 23.75% had mild mood disturbance, 10.00% had borderline clinical depression, 11.25% had moderate depression, 7.50% had severe depression, and 7.50% had extreme depression. According to the Rosenberg Self-esteem scale, 30.00% had low self-esteem, 37.50% had fluctuating, and 32.50% had high self-esteem. The finding that both BDI and BAI were significant predictors of RSES, indicating that higher levels of depression and anxiety were associated with lower levels of self- esteem (RSES), is consistent with previous research that we refer to above, although comparative analysis has not been attainable as a result of the implementation of numerous evaluations and achieving processes in these research investigations. As a consequence, the emotional wellness features of Greek practicing lawyers analyzed in the present investigation are comparable to what has been reported among US lawyers [42] and Poland [60].

The second hypothesis was that there is a negative relationship between self-esteem and anxiety symptomatology among practicing lawyers. The obtained result indicates that a significant level of low and moderate stress among the Greek population of lawyers may lead to antipathy to clients, decreased focus, reduced recall and creative imagination, or heightened anger, which can be terrible for the profession of law [60]. Headaches, muscle discomfort, backache, and discomfort in the neck were common among the lawyers investigated, as were complaints from many different parts of the human organism, according to our research.

Regarding the hypothesis that depressive and anxiety symptomatology predict self-esteem among practicing lawyers, such that higher levels of symptomatology are associated with lower levels of self-esteem, it was also proven in previous studies that stress has an effect on mental health, devotion to work, and can limit motivation to take action. An adverse state of mind may weaken the immune system and, in turn, result in a decline in health, especially severe depression or workplace burnout in lawyers [55,63].

Also, our findings are similar to previous research in other groups, like patients with Polycystic ovary syndrome (PCOS) [31], individuals with cardiovascular diseases [32], young adults [33], couples undergoing infertility treatment [34], university students [35], adult acquired brain injury [36], older people in nursing homes [37], and the migrant population [38].

### 4.1. Addressing Hypotheses

The present study examined the impact of anxiety and depressive symptomatology on self-esteem among Greek practicing lawyers.

The first hypothesis proposed a negative association between self-esteem and depressive symptomatology. This was strongly supported, as depressive symptoms emerged as the most significant predictor of lower self-esteem, consistent with previous research on other high-stress professions.

Similarly, the second hypothesis, which suggested a negative association between anxiety and self-esteem, was also confirmed. Although anxiety was a less potent predictor than depression, its contribution was statistically significant, highlighting its role in shaping self-esteem among lawyers.

The third hypothesis posited that anxiety and depressive symptomatology collectively predict self-esteem. The findings validated this hypothesis, with the regression model explaining 44% of the variance in self-esteem scores. These results align with earlier studies conducted in the U.S. and Poland, underscoring the universal impact of mental health on self-esteem across legal systems.

### 4.2. Contextualizing the Findings

In the Greek context, cultural and systemic factors may amplify the observed relationships. For instance, economic instability and judiciary inefficiencies place additional pressure on lawyers, potentially exacerbating mental health issues. Unlike their counterparts in other countries, Greek lawyers face limited institutional support for mental well-being, which may further contribute to reduced self-esteem. The findings also resonate with research on other high-stress professions, such as healthcare and corporate leadership, where mental health challenges significantly impact professional performance and personal well-being. For example, studies on physicians and executives reveal similar patterns of anxiety and depression negatively influencing self-esteem, particularly in the absence of effective mental health interventions.

### 4.3. Implications for Practice

The results of the study emphasize the urgent need for targeted mental health support within the Greek legal profession. To mitigate the impact of anxiety and depression among lawyers, interventions such as resilience training, stress management workshops, and confidential counseling services are crucial. Additionally, fostering a supportive workplace culture that prioritizes mental health can significantly enhance lawyers’ self-esteem and overall well-being. Addressing mental health issues within the legal profession not only benefits individuals but also has broader implications for the legal system. Lawyers in better mental health are more likely to demonstrate improved decision-making, effective client communication, and stronger professional relationships, thereby contributing to more favorable legal outcomes.

To effectively support mental health within the legal profession, several key initiatives should be implemented:

1. Workplace Mental Health Programs: Legal organizations should offer confidential counseling services, stress management workshops, and mental health awareness campaigns. These efforts will reduce the stigma surrounding mental health and promote emotional well-being.
2. Policies for Work-Life Balance: To combat burnout and improve overall well-being, legal organizations should introduce flexible work arrangements, mandatory vacation policies, and reasonable billing targets. These measures would create a healthier work environment, allowing lawyers to better balance their personal and professional responsibilities.
3. Targeted Support for Early-Career Lawyers: Young lawyers, in particular, may benefit from tailored interventions. Mentorship programs and resilience training could provide essential guidance, help navigate professional challenges, and foster confidence in early-career lawyers.
4. Systemic Reforms: Broader systemic changes are needed to address the structural stressors within the legal profession. These include reforms to improve judicial efficiency, the establishment of standardized mental health support systems, and the introduction of economic assistance initiatives to support lawyers facing financial instability.
5. Incorporating Mental Health in Legal Education: Law schools should integrate mental health education into their curricula, offer on-campus counseling services, and cultivate a collaborative academic culture. These efforts will better prepare future lawyers for the emotional and psychological challenges of the profession.
6. Monitoring and Evaluation: Regular mental health surveys and anonymous feedback systems should be implemented to assess the effectiveness of mental health initiatives. Data-driven adjustments to programs will ensure continuous improvement in addressing the mental health needs of legal professionals.
7. Cultural Change Advocacy: Senior legal professionals should take an active role in advocating for mental health awareness within the profession. Developing peer support networks where lawyers can share experiences and coping strategies in a non-judgmental setting will further enhance support for mental well-being.

Collectively, these initiatives aim to improve mental health outcomes, reduce stress, and create a more supportive and sustainable work culture within the legal profession. By addressing these critical issues, legal organizations can foster healthier, more resilient lawyers, ultimately contributing to better legal outcomes and a more efficient legal system.

### 4.4. Limitations

There are some study limitations that are worthy of mention. First of all, the research relied on self-reported data which were not independently verified. As a result, we cannot rule out the possibility of additional underlying factors influencing the findings. Moreover, there may be a bias in the sample, as lawyers who were unable to respond to the survey may have been busier, potentially leading to an underrepresentation of individuals experiencing anxiety symptoms and an overrepresentation of those reporting depression symptoms.

Additionally, the study depends on individual self-reports, which may include biases or inaccuracies, such as social desirability bias or recall errors. Another limitation inherent in the correlational nature of the study is that it can identify relationships between variables but cannot establish causality [64]. While regression analyses can highlight associations between anxiety, depression, and well-being, they do not confirm the association is causative [65]. It could be challenging to identify whether or not any apparent changes in well-being are in relation to anxiety and depressive symptomatology. Moreover, the sample size may not have been large enough to detect statistically significant changes within certain categories, which could limit the generalizability of the results. It is important to acknowledge these limitations when interpreting the study’s findings and when considering their implications in future research.

Future studies should seek to replicate these findings with larger, more diverse samples, and longitudinal designs could provide a better understanding of the causal relationships between the identified variables.

This study represents a significant first step in exploring mental health among Greek lawyers and provides a valuable foundational understanding of this important issue. While the current sample yields meaningful insights, we see exciting opportunities for enhancement by incorporating a broader and more representative demographic. Although our initial focus was on evaluating the significance of the results without detailed demographic data, we recognize that including factors such as age and gender will enrich our analysis.

Looking ahead, a follow-up study will aim to expand on these findings by collecting comprehensive demographic information, allowing for a deeper exploration of the variables influencing mental health in this population. By broadening the research scope, we hope to achieve a more thorough understanding of the factors involved and strengthen the overall robustness of our conclusions. This foundational study paves the way for future research that can further illuminate this vital topic.

### 4.5. Future Research Propositions

One proposition for future study in the field is to examine the impact of anxiety and depressive symptomatology between newer and experienced practicing lawyers’ self-esteem, because studies frequently show that rookie attorneys are less happy than more seasoned attorneys over time [66]. Also, it would be very interesting to research the differences in impact between female and male practicing lawyers’ self-esteem, to observe how gender influences the symptomatology in lawyers [42,67].

## 5. Conclusions

Finally, it is crucial to keep in mind that lawyers are more vulnerable to depressive conditions, somatization, anxiety, and stressful situations than the rest of the population. So both anxiety and depressive symptomatology have a significant negative impact on the self-esteem of practicing lawyers, as was proven according to our hypothesis. It is necessary to construct a template of more healthy behavior amongst lawyers that takes into consideration every single one of the aspects that can impact their psychological well-being. Lawyers’ awareness of the potential health dangers associated with their work, backed up by an emotional well-being awareness campaign, would undoubtedly help to reduce the prevalence of the aforementioned diseases. Throughout regular background collection, it is always important to evaluate the patient’s psychological and behavioral condition because it can have a substantial impact on the course of treatment and therapy. The ill health of the studied group is not solely a result of the lawyers’ own actions; the effects of neglected problems, in addition to the simultaneous presence of physical and mental conditions, place a substantial strain on the healthcare system as a whole [60].

## Figures and Tables

**Table 1:** Descriptive statistics.

	Mean	SD	N
Descriptive Statistics			
RSES	27.75	5.38	80
BDI	18.01	13.85	80
BAI	12.81	12.07	80

Note: RSES = Rosenberg Self-Esteem Scale; BDI = Beck Depression Inventory; BAI = Beck Anxiety Inventory; SD = Standard Deviation; N = Sample Size.

**Table 2:** ANOVA analysis for RSES, BDI and BAI.

ANOVA	SS	df	MS	F	*p*
Regression	1014.739	2	507.370	30.659	0.000
Residual	1274.261	77	16.549		
Total	2289.000	79			

Note: SS = sum of squares; df = degrees of freedom; MS = mean square; F = F-value; *p* = *p*-value.

**Table 3:** Table of coefficients.

Regression Coefficients	B	SE	Beta	t	*p*	%CI
Constant	32.943	0.811		40.628	0.000	31.328 34.558
BDI	−0.217	0.035	−0.558	−6.217	0.000	−0.286 −0.147
BAI	−0.100	0.040	−0.225	−2.505	0.014	−0.180 −0.021

Note: B = unstandardized coefficients; SE = standard error; Beta = standardized coefficients; t = t-value; *p* = *p*-value; %CI = confidence interval.

## Data Availability

The data presented in this study are available on request from the corresponding author due to privacy.

## Appendix A

**Participant Information Sheet**
You are being invited to take part in a research study. Before you decide whether or not to take part, it is important for you to understand why the research is being done and what it will involve. Please take time to read the following information carefully.
**Study Title** **:**
The impact of anxiety and depressive symptomatology on practicing lawyers’ self-esteem
**What is the purpose of the study?**

- In the present research, we focus on the impact of anxiety and depressive symptomatology on practicing lawyers’ self-esteem. Lawyers have reported relatively higher scores in job control, psychological demands and effort, and high prevalence of self-perceived work stress.
- The study is concerned with depression and anxiety symptomatology only, while data from the questionnaires do not count as an official diagnosis of depression or anxiety, and for this reason feedback from individual scores will not be provided. The information obtained will be used only for research purposes.
- The research is conducted by a student as part of BSc (hons) Psychology dissertation at the University of Sunderland.

**Why have I been approached?**

- You have been approached because you are a practicing lawyer, fluent in English and you are over 18 years of age of any gender identity, ethnicity and nationality.
- The target sample size of this research is approximately 80 individuals.

**Do I have to take part?**

- No, you do not have to participate. Participation in the study is entirely voluntary and there will be no consequences if you decide not to participate or to withdraw at a later stage.
- You will be asked to complete a consent form before participating in the study.
- During the study you may skip any questions you are not happy to answer.
- Anyone who is currently suffering from clinically diagnosed anxiety or depression should not participate in this study.

**What will happen if I don’t want to carry on with the study?**

- You have the right to change your mind and withdraw from the study without giving a reason and without incurring any penalties by simply closing your web browser and not submitting your answers. All data collected up to the point of withdrawal will be immediately destroyed.
- As this is an online anonymous study, however, data cannot be deleted after your participation has been completed, as the researcher will not be able to track specific data back to specific participants.
- For any further information please contact the experimenter through the information provided at the end of this sheet.

**What will happen to me if I take part?**
The research will consist of three questionnaires: the Beck Anxiety Inventory (BAI), which consists of 21 self-reported items to assess the intensity of anxiety symptoms during the past week, the Beck Depression Inventory (BDI), which contains 21 questions regarding your mood during the past two weeks, and the Rosenberg Self-esteem scale (RSES), a 10-item scale that measures positive and negative feelings about the self.
Demographic data will also be questioned at the beginning, such as age and gender. The entire survey will take approximately 15 min to be completed.
**What are the possible disadvantages and risks of taking part?**

- There are no particular disadvantages or risks involved in taking part in this study. However, there is a possibility that you may feel distress while answering questions about anxiety or depressive symptomatology.
- For this reason, please remember that you have the right to withdraw at any time.
- In case of psychological distress, you can contact the experimenter or her supervisor using the details provided at the end of this sheet.
- If you feel that you require additional support, please see the details for the local mental health center in Thessaloniki: Karaoli and Dimitriou Kyprios 1, Thessaloniki 546 30, Telephone: 231 331 0700.

**What are the possible benefits of taking part?**

- There are no direct benefits of taking part in the research.
- Participation will help to increase knowledge in the topic area.

**What if something goes wrong?**
If you are unhappy with the conduct of this study please contact my supervisor, Melissa Theocharidou, or the Chair of the University of Sunderland Research Ethics Group, Dr John Fulton (john.fulton@sunderland.ac.uk).
**How will my information be kept confidential?**

- All participant information will be treated according to EU GDPR (2016). All data will be completely anonymous and no personal information will be asked of you (name, email, etc.) so that data cannot be traced back to you. My supervisor and I will have access to the data. They will be kept on password-protected computers and files and will be deleted after the dissertation has been graded.
- Completely anonymised data from the project may be shared with other researchers and/or used for teaching purposes.
- The data may be looked at by staff authorised by the University of Sunderland for audit and quality assurance purposes.

**What will happen to the results of this study?**

- Results will be written up in my project report and project poster. If suitable, results may be published in academic journals and/or presented at academic conferences.

**Who is organising and funding the research?**
The research is organised by Andreas-Nikolaos Koukoulis, who is a BSc Psychology student at the University of Sunderland, Faculty of Health Sciences and Wellbeing, School of Psychology.
This project is not externally funded.
**Who has reviewed the study?**
The study has been reviewed and approved by the University of Sunderland Research Ethics Group’s review system.
**Further information and contact details**

- Andreas-Nikolaos Koukoulis
  Email: ankoukoulis@deicollege.gr
- Melissa Theocharidou (Research Supervisor)
  Email: mtheocharidou@deicollege.gr

**Thank you for taking time to read the information sheet**!
